# Editorial: Integrative approaches to acute brain injury: vascular, electrical, and metabolic interactions

**DOI:** 10.3389/fphys.2026.1944693

**Published:** 2026-08-18

**Authors:** Sérgio Brasil, Davi Jorge Fontoura Solla, Ryan Hoiland

**Affiliations:** 1 Division of Neurosurgery, Department of Neurology, School of Medicine University of São Paulo, Sao Paulo, Brazil; 2 Faculty of Medicine and Department of Cellular and Physiological Sciences at the University of British Columbia, Vancouver, BC, Canada

**Keywords:** acute brain injury, cerebral perfusion pressure, cerebrovascular autoregulation, intracranial compliance, intracranial pressure, neuromonitoring

## Background

For decades, neurocritical care management for severe neurological conditions like traumatic brain injury (TBI) has revolved around uniform, population-averaged thresholds. Standard practices, such as those outlined by the Brain Trauma Foundation, prioritize keeping intracranial pressure (ICP) below 20 or 22 mmHg and maintaining cerebral perfusion pressure (CPP) within a static 60–70 mmHg range (Brain Trauma et al., 2007). While these static markers have streamlined clinical guidelines, long-term patient outcomes have seen limited improvement. This gap exists because population-based parameters fail to account for patient-specific phenotypes, injury heterogeneity, and the highly dynamic nature of individual cerebral physiology. This Research Topic highlights a definitive paradigm shift away from a “one treatment fits all” approach and toward personalized, autoregulation-oriented, and non-invasive frameworks for tracking intracranial dynamics.

## Continuous personalized metrics and autoregulation

The foundation of individualized physiology is comprehensively detailed by Stein et al. (2025), who review how cerebrovascular reactivity (CVR) optimization can be leveraged to derive dynamic, real-time targets at the patient bedside (Stein et al., 2025). Moving past fixed population constraints, continuous metrics such as optimal cerebral perfusion pressure (CPPopt), optimal mean arterial pressure (MAPopt), individualized ICP thresholds (iICP), and optimal depth of sedation (BISopt) are tailored to a patient’s immediate physiological needs. These tools shift the treatment focus to maintaining patients within the precise pressure zones where their cerebral vessels can actively self-regulate. However, as the authors note, widespread clinical translation requires addressing current limitations, such as proprietary algorithmic lack of transparency, signal artifacts, and the need for robust validation outside of highly curated datasets.

## Redefining non-invasive hemodynamic monitoring

To reliably manage these personalized targets, clinicians must understand the physical mechanisms driving vascular and waveform behaviors. Addressing this need, Schaafsma (2025) introduces the “Theory of Arterial Acceleration” (AA), offering a novel perspective for interpreting transcranial Doppler (TCD) monitoring in severe TBI (Schaafsma, 2025). The theory proposes that myocardial contraction is actively supported by a short-lasting peristaltic contraction of arterial smooth muscle, generating the initial systolic peak (Sys1) seen in velocity waveforms (Schaafsma, 2025). Crucially, during periods of intracranial hypertension, this Sys1 component demonstrates superior tissue penetration compared to the subsequent cardiac volume-driven peak (Sys2) or diastole. This framework provides a clear physiological explanation for variations in the TCD pulsatility index (PI) and shows how brief, intermittent TCD waveform analysis can directly capture regional brain perfusion, avoiding the logistics and discomfort of continuous head-frame monitoring (Schaafsma, 2025).

## Non-invasive compliance and compensatory reserve

A non-invasive point-of-care approach showed that key physiological properties—MAPopt, intracranial compliance (ICC), and compensatory reserve—can be assessed in real-world clinical settings (Brasil et al., 2025). Utilizing an external skull microdynamics sensor to detect micrometric cranial expansions, they demonstrate that non-invasive surrogate ICP waveform features—specifically the P2/P1 ratio and time-to-peak (TTP)—satisfactorily correlate with the established, invasive amplitude-pressure index (RAP index) (Brasil et al., 2025).

Remarkably, their clinical findings show that the exhausted compensatory reserve zone corresponded to a median ICP of 19.45 mmHg. This revelation directly challenges traditional standard targets, proving that critical buffering capacity exhaustion can occur *below* the conventional 22 mmHg thresholds typically used to trigger aggressive therapeutic escalation. Additionally, the authors identify a distinct “ceiling effect” where pulse deformation becomes non-linear during total reserve depletion, reinforcing that a multimodal approach combining absolute values with wave morphology is essential to prevent clinical misinterpretation (Brasil et al., 2025).

## Integrating intracranial dynamics for the future

The ultimate success of personalized medicine in neurocritical care depends on combining these diverse physiological insights. By transitioning from isolated numeric thresholds to an integrated view of intracranial dynamics—uniting the automated CVR target optimization described by Stein et al., the precise peripheral vascular logic of Schaafsma’s AA theory, and the predictive bedside compliance wave diagnostics validated by Brasil et al.—clinicians may identify and treat ICC deterioration before life-threatening crises occur. This Research Topic demonstrates that custom-tailored hemodynamic management and reliable point-of-care toolsets are no longer experimental concepts, but the foundational standard for the next generation of critical care.
